# Insights into the DHQ-BN: mechanical, electronic, and optical properties

**DOI:** 10.1038/s41598-024-52347-2

**Published:** 2024-01-30

**Authors:** K. A. Lopes Lima, F. L. Lopes Mendonça, W. F. Giozza, R. T. de Sousa Junior, L. A. Ribeiro Junior

**Affiliations:** 1 Institute of Physics, University of Brasília, Brasília, 70910-900 Brazil; 2 Computational Materials Laboratory, LCCMat, Institute of Physics, University of Brasília, Brasília, 70910-900 Brazil; 3 Department of Electrical Engineering, Faculty of Technology, University of Brasília, Brasília, Brazil

**Keywords:** Materials science, Nanoscale materials, Theory and computation

## Abstract

Computational materials research is vital in improving our understanding of various class of materials and their properties, contributing valuable information that helps predict innovative structures and complement empirical investigations. In this context, DHQ-graphene recently emerged as a stable two-dimensional carbon allotrope composed of decagonal, hexagonal, and quadrilateral carbon rings. Here, we employ density functional theory calculations to investigate the mechanical, electronic, and optical features of its boron nitride counterpart (DHQ-BN). Our findings reveal an insulating band gap of 5.11 eV at the HSE06 level and good structural stability supported by phonon calculations and ab initio molecular dynamics simulations. Moreover, DHQ-BN exhibits strong ultraviolet (UV) activity, suggesting its potential as a highly efficient UV light absorber. Its mechanical properties, including Young’s modulus (230 GPa) and Poisson’s ratio (0.7), provide insight into its mechanical resilience and structural stability.

## Introduction

Ever since the groundbreaking synthesis of graphene in 2004 by Novoselov et al.^1^, extensive research efforts have been devoted to exploring the vast potential of two-dimensional (2D) materials, seeking to exploit their unique optoelectronic, structural, and thermal properties for applications in flat electronics^2–4^. Graphene’s exceptional attributes have opened doors to new possibilities in nanotechnology^5–8^. However, its intrinsic zero-bandgap nature presents limitations for specific optoelectronic applications, prompting the exploration of new 2D materials capable of addressing this challenge^9–14^.

To fill this gap, both theoretically and experimentally, researchers have been actively engaged in identifying 2D materials with tailored bandgaps that are conducive to optoelectronic applications, such as solar cells and light-emitting diodes^15,16^. Promising materials emerging from these efforts include transition metal dichalcogenides^17^, black phosphorus (phosphorene)^18^, boron nitride monolayers^19^, and various hybrid organic-inorganic perovskite structures^20^, each possessing distinct electronic properties, tunable bandgaps, and exceptional optoelectronic characteristics.

In computational materials science, quantum, and classical approaches have become indispensable for materials modeling and design, often preceding the synthesis of novel nanostructured materials by decades^21–24^. Recent advances in material modeling have paved the way for the theoretical exploration of 2D materials of boron nitride, which exhibit topological similarities to their carbon-based counterparts^25–30^. Some examples of these boron nitride structures are hexagonal boron nitride (h-BN)^31^, binary monolayer amorphous boron nitride^32^ (MABN), pentagraphene-like BN (penta-BN)^32^, T-graphene-like BN (T-BN)^33^, phagraphene-like BN (pha-BN)^34^, and the BN biphenylene network (BN-BPN)^35–38^.

In this context, DHQ-BN, the counterpart to boron nitride of DHQ-graphene^39^, stands out as a solution to the challenge of zero-band-gap limitations in optoelectronic applications. Its potential lies in enabling to more effective light absorption and emission within specific energy ranges. DHQ-graphene, a recently discovered two-dimensional carbon allotrope characterized by a unique arrangement of decagonal, hexagonal, and quadrilateral carbon rings, has garnered attention. So far, DHQ-BN’s structural, mechanical, electronic, and optical properties remain untested.

DHQ-graphene is a metastable material exhibiting anisotropic mechanical properties. Its in-plane Young’s modulus is comparable to that of penta-graphene^32^. DHQ-graphene demonstrates metallic behavior with a significantly higher electronic density of states, approximately 0.193 eV per state per atom. The bridge site at the edge shared by six- and ten-membered rings in DHQ-graphene is the most favorable adsorption site, displaying adsorption energy for oxygen (− 0.27 eV). A charge transfer of 0.834*e* from a DHQ-graphene sheet to an O atom, 3.35% higher than the one for perfect graphene, suggests its potential for controlled oxygen adsorption.

The nomenclature of 2D carbon and boron nitride allotropes has become significant due to their growing diversity and intricacy^40^. Ensuring clarity and scientific precision in the characterization of these materials is essential^40^. Hence, we have retained the original name of the carbon-based structure under study (the DHQ-graphene) while referring to its boron nitride variant with analogous topology.

This study used density functional theory (DFT) calculations to thoroughly examine DHQ-BN, delving into its mechanical, electronic, and optical properties. We establish the material’s structural stability by observing an absence of negative frequencies in the phonon spectrum. Moreover, the material exhibits exceptional thermal stability, as evidenced by its lack of topology reconfiguration at 1000 K in ab initio molecular dynamics (MD) simulations. The results also unveil the direct bandgap of DHQ-BN at 5.11 eV and its significant optical absorption capacity within the ultraviolet (UV) range. We explored its mechanical behavior, which varies depending on the applied strain direction, yielding Young’s Modulus values ranging from 30 to 230 GPa.

## Results and discussion

**Figure 1:**
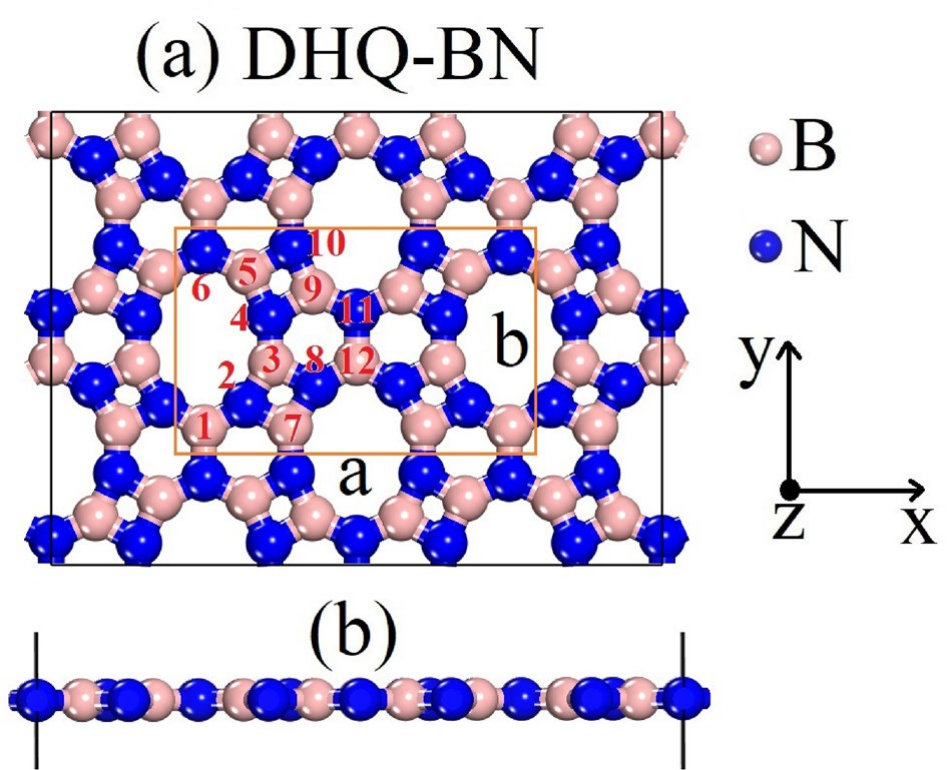
Illustration depicting the DHQ-BN topology, presented from both a top view (**a**) and a side view (**b**). Emphasized within the diagram is the unit cell, with boron and nitrogen atoms represented by the colors pink and blue, respectively. The red numbers pinpoint the atoms responsible for forming distinct B-N bonds.

We begin by analyzing the optimized lattice arrangement of DHQ-BN, as illustrated in Fig. 1. Geometry optimization calculations, performed with the PBE and HSE06 methods, produce comparable lattice parameters. However, for the sake of this discussion, we focus on the results obtained using the HSE06 method, which is highlighted in Fig. 1 and summarized in Table 1. The lattice vectors *a* and *b* are determined to be 9.13 Å and 6.78 Å, as indicated in Fig. 1. The crystal structure of DHQ-BN aligns with the P2/C (C2H-4) space group, pointing to its orthorhombic nature.

**Table 1:** Bond distances for the atoms highlighted in Fig. 1 calculated at the HSE06 level.

Bond type	Bond (Å)	Bond type	Bond (Å)
B1-N2	1.41	B3-N4	1.41
B2-N7	1.48	B3-N8	1.48
B7-N2	1.48	B5-N4	1.47
B5-N6	1.45	B5-N10	1.50
B9-N4	1.51	B9-N10	1.47
B9-N11	1.45	B12-N8	1.40

**Figure 2:**
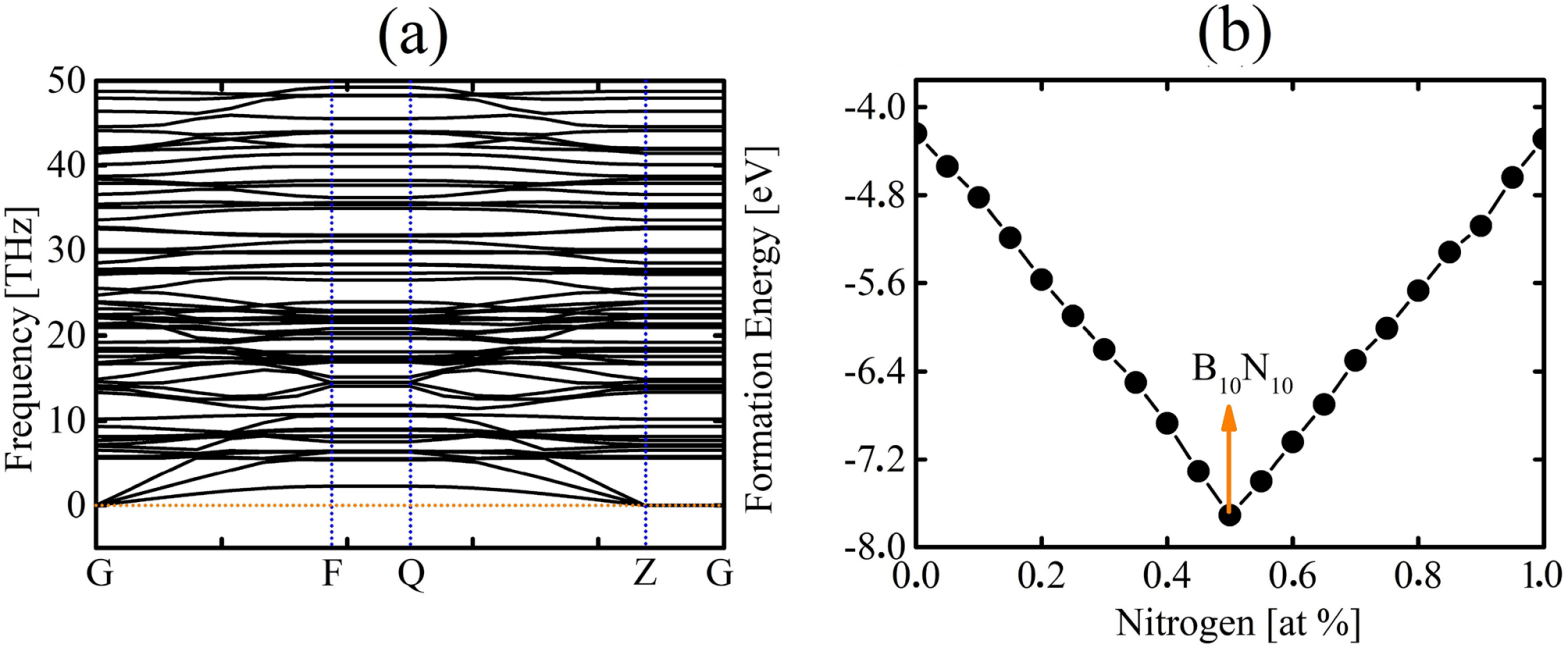
(**a**) Phonon dispersion and (**b**) computed formation energy as a function of the nitrogen concentration for DHQ-BN, calculated at PBE level.

Figure 2a illustrates the dispersion of phonons in the DHQ-BN network. No imaginary frequencies are present, suggesting the material’s dynamic stability. The absence of a band gap between the acoustic and optical modes indicates a significant scattering rate and brief phonon lifetime, contributing to the material’s moderate lattice thermal conductivity. Furthermore, the formation energy of DHQ-BN is − 7.71 eV.

When the formation energy of a structure aligns with the convex hull, it signifies thermodynamic stability and feasibility for experimental realization. We have pinpointed a unique global minimum structure within this two-dimensional parameter space by analyzing the relationship between formation energy and nitrogen concentrations, as illustrated in Fig. 2b. This specific minimum is situated precisely at the midpoint, characterized by an N:B ratio of 1:1. This composition resembles a structure reminiscent of the all-carbon DHQ-BN.

The convex hull plot was generated by systematically varying the concentration of N in the structure that was initially composed solely of B atoms while keeping the overall composition constant. This allowed us to assess the energetic stability of different configurations with distinct proportions between B and N atoms. As a result, one can note that the most favorable structure for the proposed lattice is indeed the one with B_10_N_10_ proportion.

We performed ab initio molecular dynamics (AIMD) simulations to assess the material’s dynamic and thermal behavior. Spanning a timeframe of 5000 fs, these simulations subjected the material to an elevated temperature of 1000 K. The selected time frame was deemed sufficient for our purposes, given the high-temperature regime adopted in our simulations. Our primary focus during these simulations was the continuous monitoring of the temporal evolution of the total energy per atom, a critical indicator of thermal stability. Employing a supercell measuring $2 \times 2 \times 1$, containing 80 atoms, we scrutinized the fluctuations in energy.

**Figure 3:**
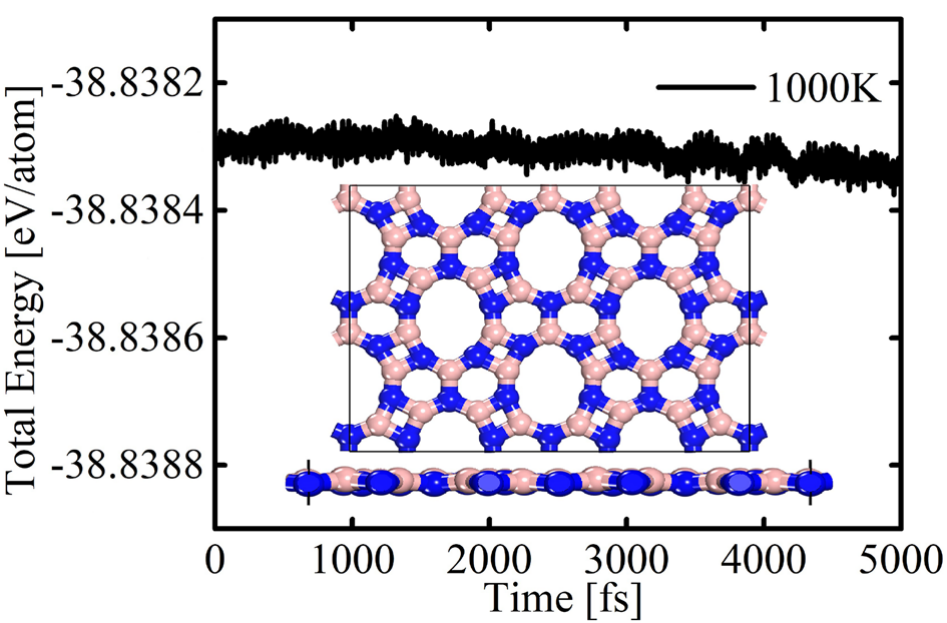
The evolution of the total energy per atom within the DHQ-BN lattice was tracked during AIMD simulations conducted at 1000 K, employing the PBE computational method. To visually represent the lattice arrangement of DHQ-BN at 5000 fs, we present insets displaying its top and side views.

The results, showcased in Fig. 3, unveil that the DHQ-BN lattice presents good thermal stability. The total energy exhibits only minimal fluctuations during the simulation, underscoring the material’s ability to maintain a balanced energy distribution. The insets in the figure provide a closer look at the DHQ-BN topology at the end of the simulation. One can note that no planarity deviations are noted. Importantly, the DHQ-BN does not entail bond breakage or reconstruction at 1000 K. The lattice pattern at 1000 K mirrors that of the optimized structure (refer to Fig. 1), reinforcing the assertion of DHQ-BN’s good thermal stability.

**Figure 4:**
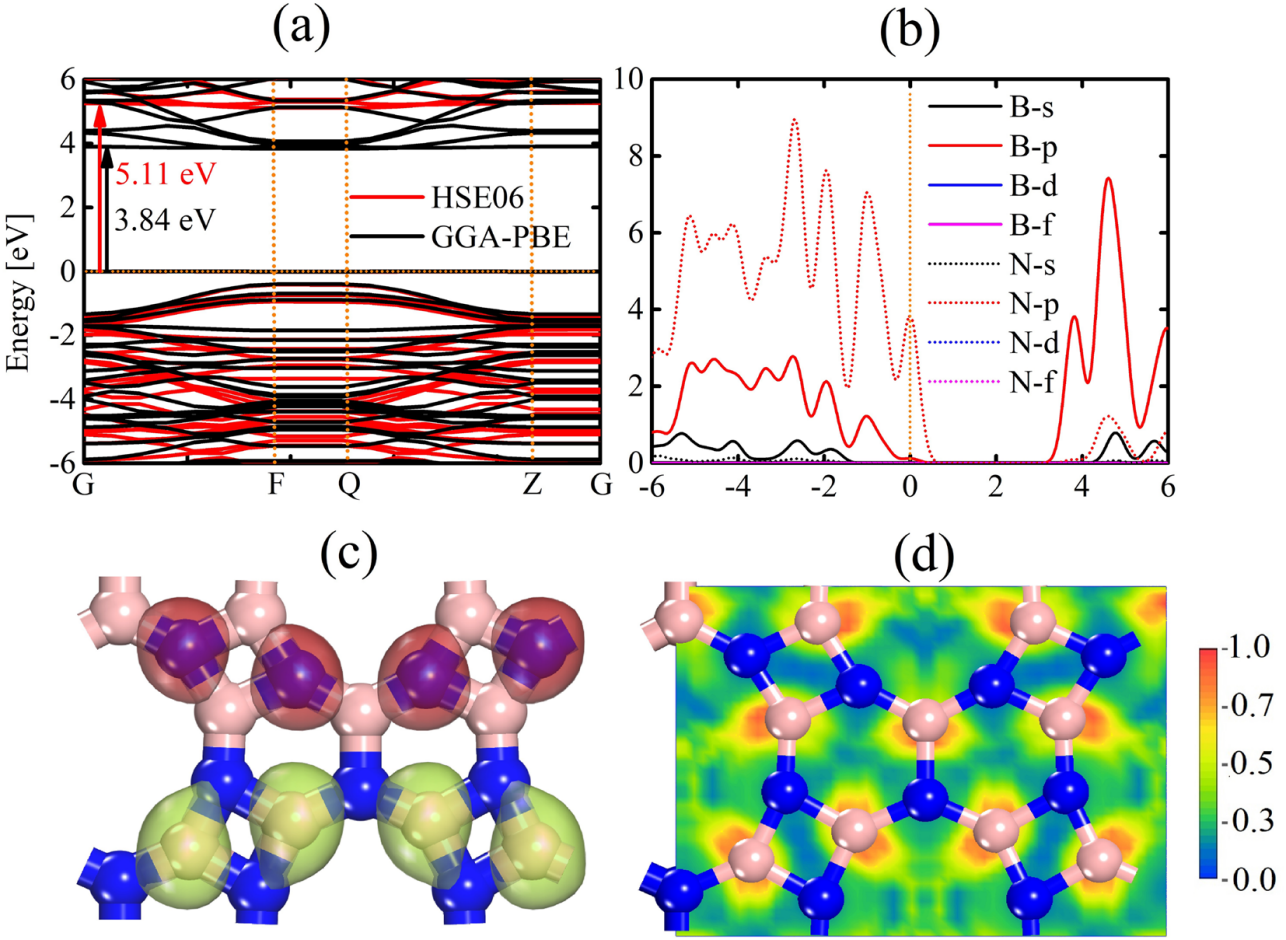
In (**a**), we present the electronic band structure, while (**b**) provides insight into the partial density of states (PDOS) within the DHQ-BN monolayer. The band structures were computed using the PBE (in black) and HSE06 (in red) methods. The PDOS analysis in (**b**) was conducted at the HSE06 level. (**c**) Illustrates the highest occupied crystal orbital (HOCO, in red), and the lowest unoccupied crystal orbital (LUCO, in green). (**d**) Schematically depicts the electron localization function. These properties were also calculated using the HSE06 approach.

Turning our attention to the electronic properties, Fig. 4a depicts the band structure profiles generated through the PBE (in black) and HSE06 (in red) methods. These calculations yield band gap values of 3.84 eV and 5.11 eV for the PBE and HSE06 levels, respectively. It is important to note that PBE calculations tend to underestimate band gap values, necessitating the adoption of the HSE06 hybrid functional to determine DHQ-BN’s electronic and optical characteristics precisely. The HSE06 band structure establishes DHQ-BN as an insulator, with energy bands near the Fermi level displaying dispersion. This dispersion signifies the delocalized nature of electronic states within the material.

It is important to note that variations in the predicted band gap values for DHQ-BN arise from the inherent limitations and approximations of computational methods. The HSE06 approach, classified as a hybrid functional, introduces a fraction of exact exchange in combination with the exchange-correlation functional. The inclusion of non-local exchange interactions serves to improve the precision of electronic structure predictions, especially in cases involving substantial electron localization or delocalization, such as semiconductors. Therefore, the band gap determined through HSE06 is frequently regarded as offering a more accurate portrayal of electronic properties.

Similarly, h-BN is characterized by an insulating band gap of approximately 5.95 eV^41^. The comparatively smaller band gap observed in DHQ-BN, compared to h-BN, can be attributed to its unique ring topology. These factors introduce distinct pathways for electronic transitions, contributing to the altered electronic properties of DHQ-BN.

It is important to acknowledge that variations in the predicted band gap values for DHQ-BN are rooted in computational methodologies’ inherent limitations and approximations. The HSE06 approach, characterized by its hybrid functional nature, incorporates a fraction of exact exchange and the exchange-correlation functional. Incorporating non-local exchange interactions significantly improves the precision of electronic structure predictions, especially for systems where electron localization or delocalization plays a crucial role, such as semiconductors. As a result, the band gap computed using the HSE06 method is frequently regarded as a more dependable representation of electronic properties.

In Fig. 4b, we delve into the partial density of states (PDOS) within DHQ-BN, employing the HSE06 hybrid functional for precise analysis. One can note a prevalence of p-states of nitrogen in the electronic structure. This prominence of p-orbitals indicates their pivotal role in driving electronic transitions and interactions within the material. They are often associated with directional bonding phenomena.

Further insights into chemical interactions within DHQ-BN are provided through the examination of the highest occupied crystal orbital (HOCO), lowest unoccupied crystal orbital (LUCO), and electron localization function (ELF) analyses, as illustrated in Fig. 4c,d. These analyses reveal distinctive orbital localizations within DHQ-BN. In Fig. 4c, one can note that HOCO predominantly localizes on nitrogen atoms of the tetragonal rings. At the same time, the LUCO primarily resides in boron atoms in the tetragonal rings. This trend is consistent with the ELF pattern, as discussed below, once there is a pronounced electron localization/delocalization on boron/nitrogen atoms. The electronegativity disparity between nitrogen and boron atoms results in an induced dipole towards the former, favoring the localization of LUCO on them. The electron localization function (ELF) assigns a numerical value between 0 and 1 to every point within space, allowing us to gain insights into the nature of electron interactions. Typically, regions where ELF values approach 1 indicate the presence of strong covalent interactions or lone pair electrons. Conversely, lower ELF values, around 0.5, suggest delocalization, potentially pointing to ionic bonds or weak Van der Waals interactions. The ELF map in Fig. 4d highlights the prevalence of localized electrons on boron atoms and delocalized electrons on nitrogen atoms, aligning with the earlier discussed charge distribution concept.

**Figure 5:**
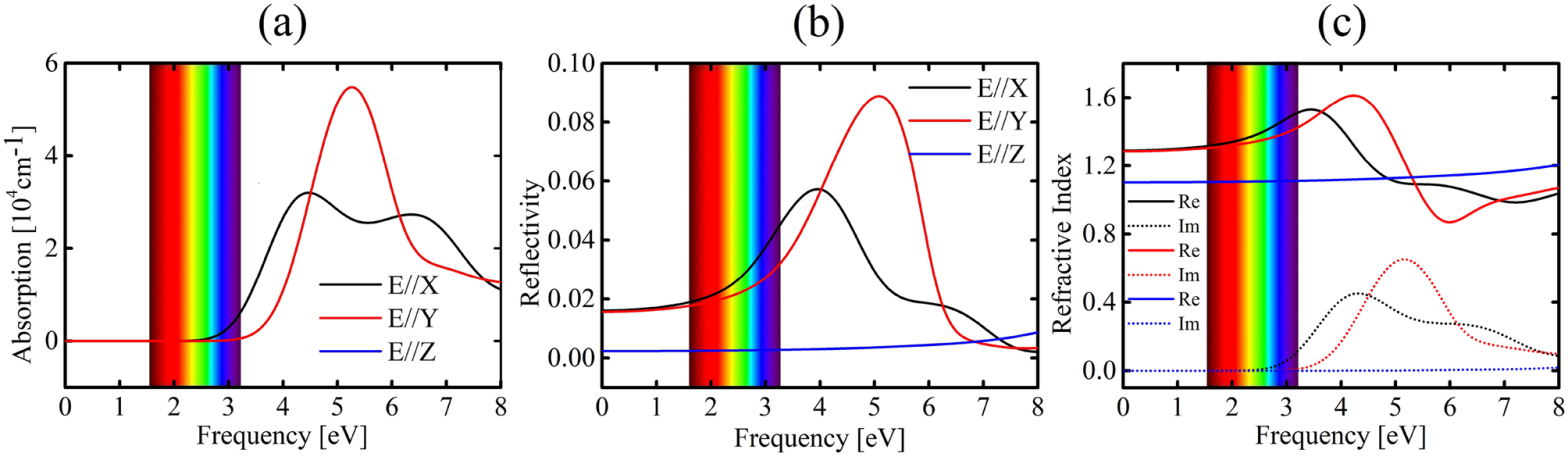
(**a**) absorption coefficient ($\alpha$), (**b**) reflectivity, and (**c**) Refractive index for polarized light along the x (E//X), y (E//Y), and z (E//Z) directions at the HSE06 level are depicted in this figure. The solid and dashed lines represent the dielectric function’s real (Re) and imaginary (Im) components.

Figure 5 provides insights into the optical properties of the DHQ-BN lattice, explicitly focusing on the polarization of light along the x (E//X), y (E//Y), and z (E//Z) directions. These calculations were carried out utilizing the HSE06 method. The solid and dashed lines in this figure correspond to the dielectric function’s real (Re) and imaginary (Im) parts. The optical properties of DHQ-BN are marked by strong optical activity, predominantly in the UV region. This characteristic aligns with the behavior observed in h-BN, which exhibits optical activity across a broad range of wavelengths, including UV and visible light^41^.

It is important to note that the band gap of h-BN typically falls within the UV range, rendering it an insulator with limited electronic conductivity. The transparent nature of both DHQ-BN and h-BN can be attributed to their insulating band gaps, preventing the absorption of photons with energies below their respective band gap values.

The DHQ-BN optical absorption is substantial and limited to the UV region, as shown in Fig. 5a. These optical characteristics suggest that this material could serve as a UV collector. DQH-BN’s low reflectivity is primarily confined to the UV region, as Fig. 5b depicts. Similarly to h-BN, DHQ-BN exhibits relatively high refractive index values, as seen in Fig. 5c. This trend indicates its ability to polarize in response to external electric fields, implying potential applications in electronics and photonics.

The elastic properties of DHQ-BN play a pivotal role in understanding microcrack behavior and its overall durability. To evaluate the anisotropy inherent in its mechanical characteristics, Poisson’s ratio ($\nu (\theta )$) and Young’s modulus ($Y(\theta )$) were determined under pressure within the xy plane, as defined by the following equations^42,43^:

1 $$\begin{aligned} \displaystyle Y(\theta ) = \frac{{C_{11}C_{22} - C_{12}^2}}{{C_{11}\alpha ^4 + C_{22}\beta ^4 + \left( \frac{{C_{11}C_{22} - C_{12}^2}}{{C_{44}}} - 2.0C_{12}\right) \alpha ^2\beta ^2}}, \end{aligned}$$

and

2 $$\begin{aligned} \displaystyle \nu (\theta )= \frac{{(C_{11} + C_{22} - \frac{{C_{11}C_{22} - C_{12}^2}}{{C_{44}}})\alpha ^2\beta ^2 - C_{12}(\alpha ^4 + \beta ^4)}}{{C_{11}\alpha ^4 + C_{22}\beta ^4 + \left( \frac{{C_{11}C_{22} - C_{12}^2}}{{C_{44}}} - 2.0C_{12}\right) \alpha ^2\beta ^2}}. \end{aligned}$$

In this context, $\alpha =\cos (\theta )$ and $\beta =\sin (\theta )$.

**Figure 6:**
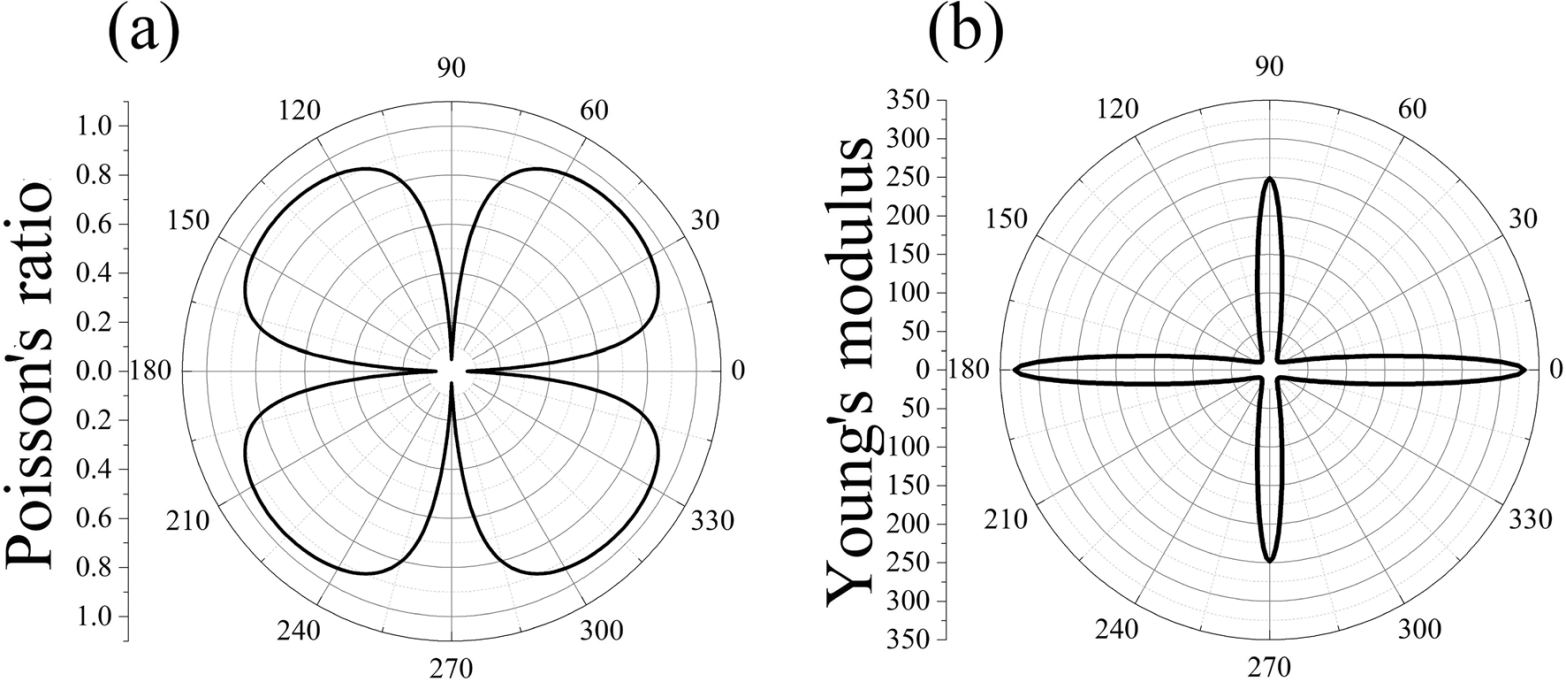
2D representation of (**a**) Poisson’s ratio and (**b**) Young’s modulus (GPa) in the xy plane for the DHQ-BN monolayer.

Table 2 presents the DHQ-BN elastic properties, and Fig. 6 shows the 2D representation of (a) Poisson’s ratio and (b) Young’s modulus (GPa) in the xy plane. Poisson’s ratio of DHQ-BN reaches a maximum value ($\nu _{MAX}$) of approximately 0.7, while the associated Young’s modulus remains below 30 GPa. This trend indicates the incompressibility of the material under biaxial strains.

**Table 2:** Elastic constants C_ij_ (GPa) and maximum values for Young’s modulus (GPa) ($Y_{MAX}$) and maximum ($\nu _{MAX}$) and ($\nu _{MIN}$) Poisson’s ratios.

Structure	C_11_	C_12_	C_22_	C_44_	$Y_{MAX}$	$\nu _{MAX}$	$\nu _{MIN}$
DHQ-BN	249.13	15.94	332.17	3.95	155.09	0.84	0.18

The anisotropic behavior of DHQ-BN is evident in its Young’s modulus values, measured at approximately 330 GPa and 230 GPa for strains along the x and y directions, respectively. These values reflect the material’s resistance to deformation under tension, with lower Young’s modulus values indicating greater deformability. The Poisson’s ratio values for uniaxial strains along the x and y directions are approximately 0.18, smaller than the h-BN one (about 0.29)^44^. The diminished Poisson ratio in DHQ-BN can be attributed to its unique structural arrangement, particularly the inclusion of decagonal rings, which enhance flexibility and extensive deformation under tension, resulting in lower Poisson ratios.

The observed anisotropy in mechanical properties in DHQ-BN stems from its unique topology. The distinctive ring distribution along different plane directions under uniaxial strain plays a crucial role. When subjected to uniaxial tensile strain in the y-direction, the aligned sequence of 10-atom porous rings facilitates easier deformation. In contrast, the tensile strain in the x-direction presents a sequence of fused hexagonal rings, making DHQ-BN stiffer in this direction. This variation in ring distribution directly influences the material’s response to mechanical stress, resulting in anisotropic mechanical behavior.

**Figure 7:**
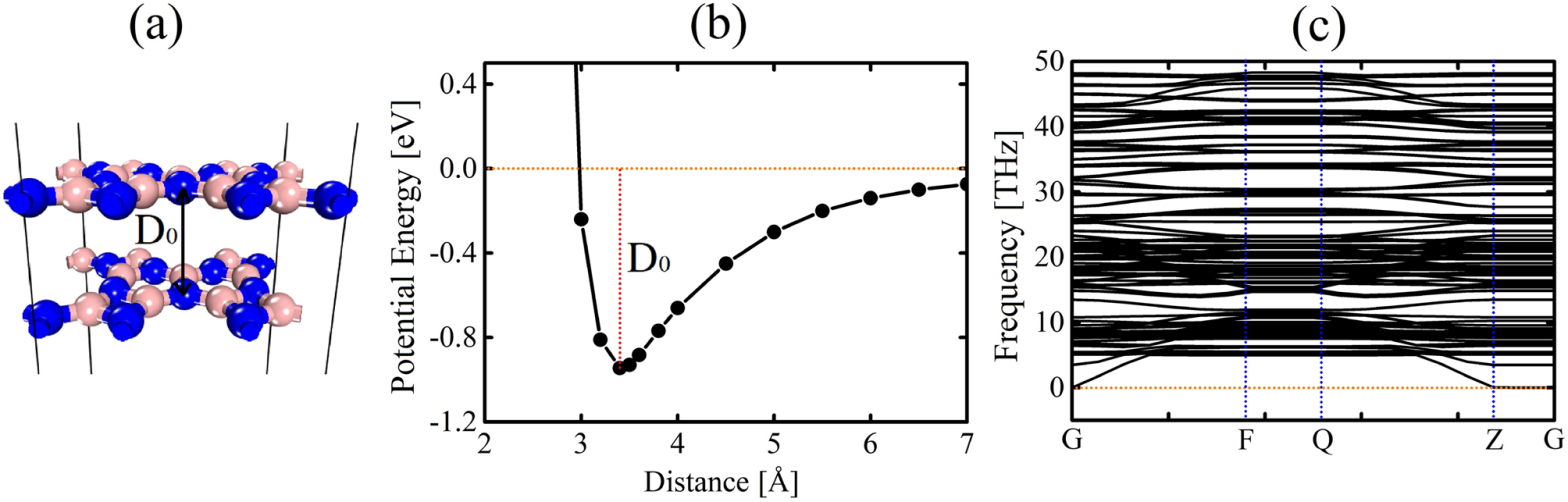
Schematic representation of (**a**) DHQ-BN AA stacking arrangement at the minimum distance, (**b**) potential energy curve as a function of interlayer distance, and (**c**) phonon spectrum for the AA-stacked configuration of DHQ-BN.

Finally, we conducted calculations on the bilayer configuration of DHQ-BN, where we observed that the minimum energy state occurred with an AA stacking arrangement, as shown in Fig. 7. Figure 7a illustrates the optimized structure of the bilayer material in the AA stacking configuration. This arrangement involves aligning the atoms in one layer directly on top of the atoms in the adjacent layer.

For the sake of comparison, we investigate here only the AA staking of DHQ-BN since previous studies consistently demonstrate the stability of the AA arrangement in bilayer h-BN systems compared to other stacking patterns^45–48^. Given this established precedence, we believe that investigating the AA stacking of DHQ-BN is sufficient to draw meaningful comparisons with its analogous h-BN.

To gain insights into the interlayer interactions and their impact on the material’s stability, we examined the potential energy curve as a function of interlayer distance for the AA-stacked configuration, as shown in Fig. 7b. We identified a specific interlayer energy, denoted as $D_0=0.95$, where the potential energy reaches its minimum value around 3.4 Å. This equilibrium distance represents the most stable separation between the two layers. It signifies that the atoms in the bilayer material stay at this specific distance due to the balance between attractive and repulsive forces.

We observed that the bilayer’s energy is higher than the monolayer’s. This energy difference highlights the significant influence of stacking arrangements on the material’s overall energy landscape. Adding the second layer introduces interactions contributing to a slightly higher energy state, emphasizing the importance of considering layer-to-layer interactions in understanding the material’s behavior.

To further assess the stability of the AA-stacked bilayer, we examined its phonon spectrum, as displayed in Fig. 7c. The absence of imaginary frequencies within the phonon spectrum indicates the stacked system’s stability. As mentioned, imaginary frequencies suggest vibrational modes that could lead to structural instabilities or phase transitions. The absence of such modes reinforces our confidence in the structural integrity of the AA-stacked bilayer.

## Methods

We conducted a series of DFT simulations to comprehensively investigate DHQ-BN’s mechanical, electronic, and optical characteristics. The lattice configuration of this material is illustrated in Fig. 1. The simulations were carried out with the CASTEP^49^ code.

Exchange and correlation effects were treated using the generalized gradient approximation (GGA), with two specific functionals applied: the Perdew–Burke–Ernzerhof (PBE)^50^ and the Heyd–Scuseria–Ernzerhof (HSE06)^51^ hybrid functional. To accurately account for the interactions among the nuclear electrons of boron and nitrogen, norm-conserving pseudopotentials tailored for these elements were employed, as integrated into the CASTEP framework.

Electronic self-consistency was achieved through the Broyden–Fletcher–Goldfarb–Shannon (BF GS) unrestricted nonlinear iterative algorithm^52,53^. We considered a plane-wave basis set with an energy cutoff of 700 eV and convergence criteria for energy as 1.0 $\times 10^{-5}$ eV. Periodic boundary conditions were applied to ensure a fully relaxed DHQ-BN lattice. The relaxation process aimed to maintain residual forces on each atom below 1.0 $\times 10^{-3}$ eV/Å, while keeping the pressure below 0.01 GPa. During lattice optimization, the basis vector in the z-direction remained fixed, and a k-grid of $10 \times 10 \times 1$ was employed. For electronic and optical calculations, k-grids of $15 \times 15 \times 1$ and $5 \times 5 \times 1$ were used for the PBE and HSE06 approaches, respectively. Additionally, Partial Density of States (PDOS) analyses were conducted at the HSE06 level, employing a k-grid of $20 \times 20 \times 1$.

Elastic properties of DHQ-BN were determined using the LDA/CA-PZ method^54,55^, and a vacuum region of 20 Å was incorporated to mitigate artificial interactions among periodic images.

It is worth noting that the choice of a reduced k-grid resolution in HSE06 calculations, compared to the PBE approach, was primarily driven by practical computational considerations, given the inherent computational expense associated with the HSE06 method. Rigorous convergence tests were conducted to validate the chosen k-grid resolution for HSE06 calculations, ensuring the required precision and reliability within an acceptable error range.

To analyze phonon properties, the linear response methodology was employed with a grid spacing of 0.05 1/Å and a convergence tolerance of 10^-5^ eV/Å^2^. For the assessment of the mechanical properties of DHQ-BN, the stress–strain method, based on the Voigt–Reuss–Hill approximation^56,57^, was utilized. Additionally, it’s noteworthy that the stability of the material was confirmed through ab initio molecular dynamics (MD) simulations, demonstrating its resistance to reconfiguration even at temperatures as high as 1000 K.

## Conclusions

In summary, our comprehensive exploration of the DHQ-BN lattice, which serves as the boron nitride counterpart to DHQ-graphene, has affirmed its structural stability. We have demonstrated the DHQ-BN dynamical and thermal stability through DFT and AIMD simulations. Our study revealed insulting band gap properties for DHQ-BN with computed values of 3.84 eV (PBE) and 5.11 eV (HSE06).

On the optical characteristics, our investigation has uncovered the lattice’s potent UV activity, hinting at its potential as an effective UV collector. The lattice’s high refractive index values have implications for applications in electronics and photonics. Examining the material’s mechanical behavior has shed light on its anisotropic traits, encompassing Poisson’s ratio and Young’s modulus. The anisotropic behavior of DHQ-BN is evident in its Young’s modulus values, measured at approximately 330 GPa and 230 GPa for strains along the x and y directions, respectively.

Possessing a broad semiconducting bandgap and electronic states with notable delocalization, DHQ-BN once manufactured can present a potential for implementation in nanoelectronics and photonics applications. Its favorable thermal stability and reduced lattice thermal conductivity position it as a candidate for addressing thermal management challenges in microelectronics. The distinctive anisotropic mechanical properties of DHQ-BN open avenues for enhancing the strength of lightweight materials. Additionally, its significant UV absorption coupled with low reflectivity positions DHQ-BN as a promising candidate for efficient UV collection, particularly relevant in sensing and detection applications.

## Data Availability

Data supporting this study’s findings are available upon reasonable request from the first author K.A.L.L.
